# Study of local intracellular signals regulating axonal morphogenesis using a microfluidic device

**DOI:** 10.1080/14686996.2016.1241131

**Published:** 2016-10-20

**Authors:** Daiki Uryu, Tomohiro Tamaru, Azusa Suzuki, Rie Sakai, Yoshiyuki Konishi

**Affiliations:** ^a^ Department of Human and Artificial Intelligent Systems, Faculty of Engineering, University of Fukui, Fukui, Japan; ^b^ Department of Materials Science and Biotechnology, Faculty of Engineering, University of Fukui, Fukui, Japan; ^c^ Life Science Innovation Center, University of Fukui, Fukui, Japan

**Keywords:** Microfluidic device, cerebellar granule neurons, axon, depolarization, dihydrofolate reductase, 60 New topics/Others, 200 Applications, 600 Applications/Cell biology

## Abstract

The establishment and maintenance of axonal patterning is crucial for neuronal function. To identify the molecular systems that operate locally to control axonal structure, it is important to manipulate molecular functions in restricted subcellular areas for a long period of time. Microfluidic devices can be powerful tools for such purposes. In this study, we demonstrate the application of a microfluidic device to clarify the function of local Ca^2+^ signals in axons. Membrane depolarization significantly induced axonal branch-extension in cultured cerebellar granule neurons (CGNs). Local application of nifedipine using a polydimethylsiloxane (PDMS)-based microfluidic device demonstrated that Ca^2+^ entry from the axonal region via L-type voltage-dependent calcium channels (L-VDCC) is required for branch extension. Furthermore, we developed a method for locally controlling protein levels by combining genetic techniques and use of a microfluidic culture system. A vector for enhanced green fluorescent protein (EGFP) fused to a destabilizing domain derived from *E. coli* dihydrofolate reductase (ecDHFR) is introduced in neurons by electroporation. By local application of the DHFR ligand, trimethoprim (TMP) using a microfluidic device, we were able to manipulate differentially the level of fusion protein between axons and somatodendrites. The present study revealed the effectiveness of microfluidic devices to address fundamental biological issues at subcellular levels, and the possibility of their development in combination with molecular techniques.

## Graphical Abstract

**Figure:**
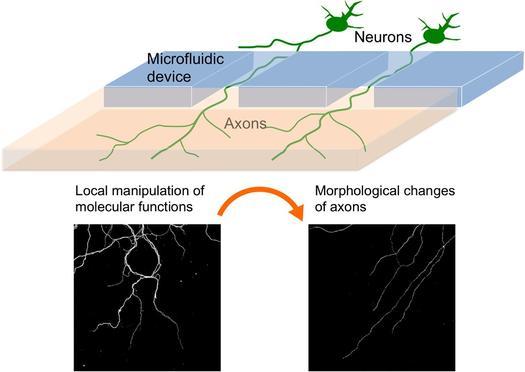

## 1. Introduction

The branched axonal morphology of neurons is important for information processing, and is constructed not only during development, but also in the adult brain.[1,2] The mechanisms by which neurons locally control their axonal morphology remain to be elucidated. Previous studies have shown that localized calcium (Ca^2+^) transients promote axonal branch extension in cortical neurons.[3] In cerebellar granule neurons (CGNs), Ca^2+^ entry via L-type voltage-dependent Ca^2+^ channels (L-VDCC) (upon depolarization induced by treatment with a high concentration of KCl) increases the growth and branching of neurites.[4] It is suggested that Ca^2+^ entry regulates dendritic maturation of CGNs via activation of multiple transcriptional machineries.[5–7] For example, neuronal transcriptional factors, NeuroD1/2 respond to calcium via phosphorylation on their transcriptional activation domain, which is required for the depolarization-dependent dendritic growth of CGNs.[5,8,9] However, the effect of membrane depolarization on CGN axonal morphogenesis remains largely unknown.

To investigate local signalling, it is crucial to manipulate the function of molecules in a region-dependent manner. Light-induced systems have been used for this purpose. One approach is to use specific compounds called caged ligands, which are activated by light.[10] More recently, optogenetic approaches in which exogenous genes for light-sensitive molecules are introduced into cells have been developed.[11] This technique has been used to manipulate neuronal activity by using light-sensitive ion channels,[11] as well as intracellular signalling and transport by light-dependent dimerization molecules.[12,13] However, since light-induced approaches can cause phototoxicity, their application to investigate long-term events such as axonal morphogenesis has been limited.

Microfluidic devices can be powerful platforms to study the local signalling involved in neuronal morphogenesis. Glass is optically transparent, thus has been widely used as a microfluidic device material.[14] However, its gas-impermeable feature hinders the long-term culture of primary neurons. Polydimethylsiloxane (PDMS) is commonly used as a material for cell culture device because of multiple advantages including high gas permeability and transparency. Taylor et al*.* [15] have developed a PDMS-based culture device to maintain central nervous system neurons long-term, by separating the axonal environment from the somatic region using microchannels^[15]^. In this study we examined the role of depolarization and local Ca^2+^ entry in axonal branch morphogenesis of CGNs using a microfluidic device. We also developed a method that allows differential regulation of protein levels between axons and somatodendrites, by combining microfluidic device and genetic techniques that enable to control protein turnover ligand dependent manner.

## 2. Materials and methods

### 2.1. CGN culture

Animals were treated according to the institutional ethical guidelines of the University of Fukui. Dissociated CGNs were prepared from Slc:ICR mice (postnatal days [P] 5–6), and placed in basal medium Eagle (BME; Sigma-Aldrich, St Louis, MO, USA) supplemented with 10% calf serum (Thermo Fisher Scientific, Waltham, MA, USA) and different concentrations of KCl, as described previously.[16] To maintain low-density cultures, neurons on glass coverslips (50–130 cells mm^–2^) were maintained as previously described.[17] Nifedipine, tetrodotoxin, and trimethoprim were purchased from Sigma-Aldrich, Abcam (Cambridge, UK), and WAKO (Osaka, Japan), respectively.

### 2.2. Microfluidic device

We used a microfluidic platform comprised of two chambers connected through microgroove arrays (10 μm × 150 μm) (AXIS; Merck Millipore, Darmstadt, Germany).[15] Devices were placed on cover glasses pre-coated with poly-L-ornithine. Approximately 1 × 10^5^ neurons were placed in the somatic chamber and maintained in media containing 10 μg ml^–1^ insulin (Sigma-Aldrich) instead of calf serum.

### 2.3. Transfection

For electroporation, CGNs (2 × 10^6^) were suspended in 100 μl of Dulbecco’s modified Eagle’s medium (Sigma-Aldrich), and mixed with plasmids. Electric pulses (160 V, 5 ms; × 2) were then applied using a T820 Electro Square Porator (BTX Molecular Delivery Systems, Holliston, MA, USA). After adding 1 ml of pre-warmed culture media, CGNs were collected by centrifugation, and resuspended in the media. When neurons were cultured in the microfluidic device, only a single electric pulse was applied. For EGFP-DD(ecDHFR) expression, a modified destruction domain of *Escherichia coli* dihydrofolate reductase (ecDHFR) [18] was introduced in the pEGFP-C vector (BD Biosciences, Franklin Lakes, NJ, USA).

### 2.4. Imaging and data analysis

Immunohistochemistry was performed as described previously.[17] To stain tubulin and F-actin, monoclonal antibody against alpha-tubulin (clone 12G10; Developmental studies hybridoma bank, University of Iowa, Iowa City, IA, USA) and rhodamine-phalloidin (Cytoskeleton Inc., Denver, CO, USA) were used. Images were obtained under an Axiovert 200 M microscope equipped with an AxioCam MRm digital camera (Carl Zeiss, Oberkochen, Germany) or confocal laser scanning microscope (LSM 5 PASCAL; Carl Zeiss). Axonal branch length was measured using ImageJ software (National Institute of Health, Bethesda, MD, USA). Compiled data are expressed as means ± standard error of the mean. Statistical analysis was done using two-tailed Student’s *t*-test.

## 3. Results and discussion

### 3.1. Depolarization enhances axonal branching in cerebellar granule neurons

**Figure 1.**
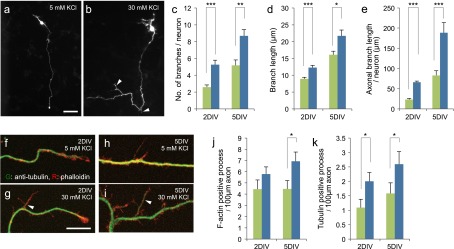
KCl-mediated membrane depolarization increases axonal branching of CGNs. (a, b) CGNs expressing EGFP were maintained in media containing 5 mM (a) or 30 mM (b) KCl. Images of neurons that were fixed at 2 DIV are shown. Arrowheads indicate axonal branches. Scale bar: 50 μm. (c–e) Quantified results obtained from CGNs at 2 and 5 DIV. The number of axonal branches (>5 μm) per neuron (c), length of each axonal branch (d), and total axonal branch length per neuron (e) were measured. For the analysis at 2 DIV, at least 45 neurons from four independent experiments were measured in each condition. For the analysis at 5 DIV, at least 24 neurons from three independent experiments were measured in each condition. (f–i) CGNs cultured at low density in media containing 5 (f, h) or 30 (g, i) mM KCl were fixed at 2 (f, g) or 5 (h, i) DIV, and subjected to immunocytochemistry using anti-tubulin antibody. To stain F-actin, neurons were simultaneously stained with rhodamine-phalloidin. Arrowheads indicate tubulin-positive processes. (j, k) Densities of phalloidin-positive (j) and tubulin-positive (k) protrusions in each condition were quantified. Experiments were performed independently three times, and at least 28 neurons in total were measured in each condition. Scale bar: 10 μm. (***p < 0.001, **p < 0.01, *p < 0.05).

To investigate the effect of membrane depolarization on axonal arborisation, primary culture CGNs expressing EGFP were maintained in media containing 5 mM or 30 mM KCl. Because of leakage of potassium through the cell membrane, treatment with a high concentration of KCl causes depolarization.[4] By two days *in vitro* (DIV), neurons in 5 mM KCl extend long axons that are distinct from other processes (Figure 1(a)). Axonal branches are rarely seen in these neurons. In contrast, axonal branch elaborations are often observed in neurons maintained in 30 mM KCl (Figure 1(b)). Quantification of the number of axonal branches (> 5 μm) revealed a more than twofold difference between the two conditions at 2 DIV (5 mM KCl: 2.6 ± 0.3, 30 mM KCl: 5.3 ± 0.5, *p* < 0.001, Figure 1(c)). Each axonal branch was slightly longer in 30 mM KCl (5 mM KCl: 8.9 ± 0.5, 30 mM KCl: 12.3 ± 0.7, *p* < 0.001, Figure 1(d)). Consequently, the total axonal branch length was higher in the 30 mM KCl compared with 5 mM KCl condition (5 mM KCl: 23 ± 3, 30 mM KCl: 66 ± 3, *p* < 0.001, Figure 1(e)). Although the overall number and length of branches increased at 5 DIV, a similar effect by KCl was observed (Figure 1(c–e)).

During the axonal collateral branch formation, short protrusions that consist of F-actin first emerge from the axonal shaft. Subsequently, microtubules extend into the short branches to form mature axonal branches. Since we did not quantify the short protrusions (<5 μm) in our initial experiments, it was not clear whether the membrane depolarization enhances the initiation of branch formation or branch maturation. To address this question, CGNs were cultivated at low density, and F-actin and tubulin in the axon were stained with phalloidin and anti-tubulin, respectively. Both tubulin-positive (arrowheads) and tubulin-negative protrusions were detected along axons under confocal microscopy (Figure 1(f–i)). The density of F-actin-positive protrusions tended to be higher in 30 mM KCl (2 DIV; 5 mM KCl: 4.4 ± 0.8, 30 mM KCl: 5.8 ± 0.6, *p* = 0.20, 5 DIV; 5 mM KCl: 4.5 ± 0.8, 30 mM KCl: 6.9 ± 0.8, *p* < 0.05, Figure 1(j)). The number of tubulin-positive processes increased over time, and was higher in the 30 mM KCl compared with 5 mM KCl condition (2 DIV; 5 mM KCl: 1.1 ± 0.3, 30 mM KCl: 2.0 ± 0.3, *p* < 0.05, 5 DIV; 5 mM KCl: 1.6 ± 0.4, 30 mM KCl: 2.6 ± 0.4, *p* < 0.05, Figure 1(k)).

These results suggest that in addition to the axonal formation, branch maturation by microtubule invasion play a role in depolarization dependent extension of axonal collateral branches in CGNs.

### 3.2. Local calcium entry via VDCC is required for the depolarization-dependent axonal branching of CGNs

**Figure 2.**
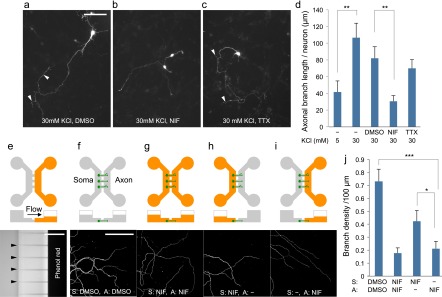
Use of microfluidic device to investigate the role of local Ca^2+^ entry in axonal branching. (a–c) CGNs (3 DIV) that were maintained in media containing 30 mM KCl for two days. Neurons were simultaneously treated with dimethyl sulfoxide (DMSO) (a), nifedipine (NIF) (b), or tetrodotoxin (TTX) (c), Arrowheads indicate axonal branches. Scale bar: 100 μm. (d), Measurement of axonal branch length. Nifedipine treatment inhibited depolarization-induced growth of axonal branches. Data in each condition were obtained from at least 36 neurons from three independent experiments. (e), Phenol red was added in the one side of chamber that has smaller volume of liquid. After 24 h, phenol red in the device was detected via UV-light absorption. Arrowheads indicate the position of microchannels. Scale bar: 100 μm. (f–i), Cerebellar granule neurons that had been maintained in a microfluidic device in the presence of 30 mM KCl were treated with nifedipine or DMSO. Reagents were applied to the somatic and/or axonal chamber as indicated. CGNs were fixed at 6 DIV and subjected to immunocytochemistry using anti-tubulin antibody. Scale bar: 50 μm. (j), Measurement of branch density along axons. Local application of nifedipine significantly inhibited axonal branching. Data in each condition were obtained from at least 49 axons from three independent experiments (***p < 0.001, **p < 0.01, *p < 0.05).

Next we asked whether Ca^2+^ entry via VDCC is involved in the regulation of axonal branching in CGNs. CGNs (1 DIV) expressing EGFP were placed in media containing nifedipine (NIF; VDCC inhibitor), tetrodotoxin (TTX; inhibitor of voltage-dependent sodium channels) or dimethyl sulfoxide (DMSO; vehicle), and incubated for a further two days (Figure 2(a–c)). Axonal branching induced by 30 mM KCl was largely inhibited by simultaneous treatment with nifedipine (DMSO: 82 ± 14, NIF: 31 ± 7, *p* < 0.01) but not with tetrodotoxin (70 ± 10, *p* = 0.47) (Figure 2(d)). These results indicate that Ca^2+^ entry via VDCC is required for the enhancement of axonal branching.

With bath application of nifedipine, it is not clear whether local Ca^2+^ entry in axons is involved in the depolarization-dependent regulation of axonal branching. To address this issue, we used CGN cultures grown in microfluidic devices that permit the fluidic isolation of axonal compartments from somatodendritic compartments.[15] We used the device that has two chambers connected by microchannels (10 μm in width, 5 μm in height and 150 μm in length). Since size of CGNs is larger than the pore size of the microchannel, they cannot move to another chamber. On the other hand, axons can path through the microchannels and reach to another side (Figure 2(e–i)). In addition, volume differences between the two chambers generate continuous flow that provides fluidic resistance that counteracts diffusion. In our experiments, volume difference between two chambers was adjusted to approximately 200 μl. As visualized in Figure 2(e) by using phenol red (molecular weight MW = 354.4), reagents can be restricted in one side of chamber.[15] By using this microfluidic system, CGNs were plated in one side (somatic side) of chambers and cultured for four days to allow axons extending in another side (axonal side) (Figure 2(f–i)). Then CGNs were treated with nifedipine (MW = 346.3), and cultured for a further two days. Consistent with the results of bath application, addition of nifedipine into both the somatic and axonal sides reduced axonal branch density (Figure 2(g) and (j)). Remarkably, local application of nifedipine into the axonal side reduced axonal branch density to a similar level as applying nifedipine in both chambers (Figure 2(i) and (j)). Application of nifedipine in the somatic side also reduced axonal branch density but was less efficient compared with treatment in the axonal side (Figure 2(h) and (j)). Thus, by using microfluidic device, we demonstrated that local Ca^2+^ entry in the axon is required for the depolarization-dependent enhancement of axonal branching.

### 3.3. Development of combined method of microfluidic device and genetic approach

As described above, the microfluidic device is useful in studying local signalling in the axon. However, to further investigate molecular systems, there is limited availability of reagents that specifically regulate the activity of molecules. Thus, we next aimed to develop the method that enables local control of the activity of various kinds of molecules. One approach to address this issue is to combine microfluidics with genetic methods. Recently, Wandless and colleagues [18,19] have demonstrated that by fusing a destabilizing domain (DD) derived from ecDHFR, it is possible to regulate protein stability in cells by adding the cell-permeable ligand trimethoprim (TMP)^[18], [19]^. In the present study, we tested whether local TMP exposure by microfluidic device is possible to manipulate expression level of DD(ecDHFR) fusion protein in restricted neuronal compartment.

**Figure 3.**
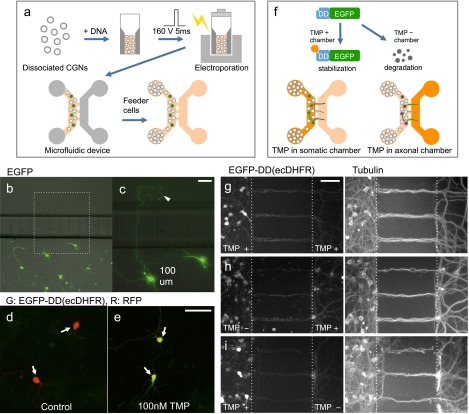
Use of microfluidic device for region-dependent manipulation of protein levels in CGNs. (a), Schematic representation of procedure for ectopic gene expression in CGNs cultivated in microfluidic device. (b, c), CGNs transfected with an EGFP expression vector were maintained in the microfluidic device and fixed at 3 DIV. A magnified image is shown in the right panel (c), Arrowhead indicates an axon extended across a microchannel. (d, e) CGNs transfected with expression vectors for EGFP-DD(ecDHFR) and RFP were treated with 0 (d) or 100 (e), nM TMP for 3 h. EGFP signal was markedly increased in the presence of TMP (arrows). (f), Schematic representation of the strategy used to locally manipulate protein levels in CGNs using a microfluidic device. (g–i), CGNs expressing EGFP-DD(ecDHFR) were maintained in the microfluidic device for seven days. TMP was applied to both the somatic and axonal chambers (g), only the axonal chamber (h), or only the somatic chamber (i), After 6 h neurons were fixed and subjected to immunocytochemistry using anti-tubulin antibody. Scale bars: 50 μm.

Firstly, we tried to efficiently express exogenous genes in CGNs maintained in the microfluidic device by electric field-mediated gene transfer. Dissociated CGNs were mixed with EGFP plasmid, a single square electric pulse (160 mV, 5 ms) was applied, and cells were then placed in the microfluidic device. To support survival of neurons that received the electric pulse, non-transfected neurons were placed in the wells of the somatic side (Figure 3(a)). In this condition, efficient neuronal survival and expression of EGFP was observed (Figure 3(b)). Furthermore, by genetically labelling neurons, the morphology of axons crossing microchannels was visualized (Figure 3(c)).

Next, we tried locally manipulating protein levels using ligand-dependent control of protein stability. Consistent with previous reports, we observed that CGNs transfected with the EGFP-DD(ecDHFR) fusion protein displayed an EGFP signal only in the presence of TMP (MW = 290.3) (Figure 3(d) and (e)).[18,19] Next, we introduced the same vector into CGNs by electroporation, and maintained the cells in the microfluidic device. At 7 DIV, TMP was applied in the somatic and/or axonal chamber, and neurons were fixed after six hours (Figure 3(f)).

We did not detect damage such as fragmentation in axonal morphology by 6 h treatment. In CGN cultures where TMP was applied in both the somatic and axonal chambers (control), EGFP-DD(ecDHFR) signal was detected in both sides, although the signal intensity was much higher in the soma than in axons (Figure 3(g)). EGFP-DD(ecDHFR) signals at the soma were weaker in CGNs that were treated with TMP only in the axonal chamber. Notably, in these CGNs, EGFP-DD(ecDHFR) signals can be detected in the axonal chamber (Figure 3(h)). In contrast, the axonal EGFP-DD(ecDHFR) signal was weak in CGNs that were treated with TMP at only the somatic chamber (Figure 3(i)). To evaluate the region dependent differences, EGFP-DD(ecDHFR) signal levels on neuronal processes in the axonal chamber were normalized by those in the somatic chamber. Compared with control CGNs, the ratio of EGFP-DD(ecDHFR) signal in the axonal chamber was significantly higher in CGNs that had TMP only in axonal side (control: 0.8 ± 0.1, TMP in axonal side: 1.9 ± 0.2, *p* < 0.01, four independent experiments). In addition, the signal in the axonal chamber in CGNs that had TMP only in the somatic side was significantly lower compared with control (TMP in somatic side: 0.5 ± 0.2, *p* < 0.001, four independent experiments).

Thus, we successfully expressed exogenous genes in CGNs cultured in a microfluidic device, and were able to differentially regulate protein levels between the somatic and axonal compartments. To achieve more precise control, the issues of protein diffusion and restriction of translation in the axon will need to be overcome.

## 4. Conclusion

We found that membrane depolarization increases axonal branching of CGNs by increasing microtubule entry into the protrusion. By using a microfluidic device, we demonstrated that both somatic and local Ca^2+^ entries through VDCCs are required for stimulation of axonal branching. By using the microfluidic device, we also developed a method for differentially regulating protein levels between the axon and somatodendrites. While other methods that use photostimulation cause neuronal toxicity, no axonal damage was observed after long-term treatment by this method. Although further improvement is required to selectively manipulate protein function in a region-dependent manner, this technique will potentially be useful for studying local signalling involved in axonal morphogenesis. In the present study, we used a device that has two compartments. In order to investigate the function of molecules in more small regions (e.g. a branch of single axon), development of microfluidic devices is anticipated. A previous study has demonstrated that local delivery of reagents in a single cell can be achieved by generating laminar flows in PDMS-based multi-channel microfluidic device.[20] However, since generation of multiple laminar flows causes a shearing stress, it is not suitable for the long-term culture of vulnerable cells such as neurons. Designing devices that enable restriction of reagents in small regions by possessing many compartments and/or micro-channels will clarify the function of local signals in neuronal morphogenesis. Furthermore, implanting electrodes in those devices would be useful for studying the relation between a local neuronal activity and downstream molecular events.

## Disclosure statement

No potential conflict of interest was reported by the authors.

## Funding

This work was supported in part by Ministry of Education, Culture, Sports, Science, and Technology [grant number 24107509].
